# Effect of genetic factors on [^18^F]FDG PET metabolic phenotypes in dementia with Lewy bodies

**DOI:** 10.1007/s00259-026-08124-6

**Published:** 2026-08-07

**Authors:** Sofie Lövdal, Sanne K. Meles, Giulia Carli, Anna Dortmond, Rosalie V. Kogan, Orly Goldstein, Mali Gana-Weisz, Rotem I. Orad, Roy N. Alcalay, Noa Bregman, Klaus L. Leenders, Tamara Shiner

**Affiliations:** 1 Department of Nuclear Medicine and Molecular Imaging, University Medical Center Groningen, University of Groningen, Groningen, the Netherlands; 2 Bernoulli Institute for Mathematics, Computer Science and Artificial Intelligence, University of Groningen, Groningen, the Netherlands; 3 Department of Neurology, University Medical Center Groningen, University of Groningen, Groningen, the Netherlands; 4 Morris K. Udall Center of Excellence for Parkinson’s Disease Research, University of Michigan, Ann Arbor, MI USA; 5 Department of Radiology, University of Michigan, Ann Arbor, MI USA; 6 Laboratory of Biomarkers and Genomics of Neurodegeneration. Tel-Aviv Medical Center, Tel-Aviv, Israel; 7 Gray Faculty of Medical & Health Sciences, Tel-Aviv University, Tel-Aviv, Israel; 8 Movement disorders Unit, Neurological Institute, Tel Aviv Medical Center, Tel Aviv, Israel; 9 Cognitive Neurology Unit, Neurological Institute, Tel-Aviv Medical Center, Tel-Aviv, Israel; 10 Sagol School of Neuroscience, Tel-Aviv University, Tel-Aviv, Israel

**Keywords:** Dementia with Lewy Bodies, GBA mutations, APOE polymorphisms, [18F]FDG PET

## Abstract

**Background:**

Neuroimaging with [^18^F]FDG PET can support the diagnosis of Dementia with Lewy Bodies (DLB), but it remains unclear how genetic factors influence metabolic phenotypes.

**Objectives:**

To determine whether *GBA1* and *APOE ε4* status are associated with diverging [^18^F]FDG PET metabolic patterns in DLB.

**Methods:**

We analyzed [^18^F]FDG PET scans from 43 patients with DLB stratified by *GBA1* and *APOE ε4* status, and 35 from healthy subjects. Analyses included the cingulate island sign (CIS), regions of interest, SSM/PCA disease patterns, and a machine learning multi-class model. We evaluated the similarity of the DLB patient scans in our cohort with respect to typical DLB, Alzheimer’s disease (AD) and Parkinson’s disease (PD)-like patterns.

**Results:**

*APOE ε4* status mainly influenced the CIS, with *APOE ε4*-negative patients showing greater preservation of the DLB-typical CIS pattern (*p* = 0.03) compared to *APOE ε4* carriers. In contrast, *GBA1* status influenced global metabolic phenotype. *GBA1* carriers showed a more homogeneous PD/DLB-like metabolic pattern in the machine learning model (*p* = 0.003) compared to *GBA1* non-carriers, whereas the latter group demonstrated greater heterogeneity and higher expression of the AD-related metabolic pattern (*p* = 0.004). These effects were observed along a metabolic spectrum rather than as distinct clusters.

**Conclusions:**

*APOE ε4* and *GBA1* modulate distinct aspects of the metabolic phenotype in DLB. *GBA1* non-carriers and *APOE ε4* carriers showed a higher rate of atypical metabolic signatures, which may contribute to biological heterogeneity and increase the risk of diagnostic misclassification.

## Introduction

 Dementia with Lewy bodies (DLB), the second most common form of neurodegenerative dementia after Alzheimer’s disease (AD), is characterized by clinical heterogeneity and mixed pathology [1, 2]. Up to three quarters of patients show Alzheimer’s disease co-pathology at autopsy [2] as well as other co-pathologies such as TDP-43 [3]. This pathological heterogeneity contributes to diagnostic uncertainty, limits the performance of standard biomarkers and complicates patient selection for clinical trials [4].

Clinically, DLB is characterized by cognitive impairment, parkinsonism, hallucinations and REM sleep behavior disorder (RBD) [1]. Although traditionally considered sporadic, genetic contributions to DLB are increasingly recognized and are likely to contribute to both clinical and biological heterogeneity. Among these, mutations in the glucocerebrosidase (*GBA1*) gene are a major risk factor across synucleinopathies, including Parkinson’s disease (PD) and DLB [5–7].

In Ashkenazi Jewish (AJ) populations, *GBA1* mutations are particularly frequent. Approximately one-third of AJ patients with DLB carry a mutation [7], making *GBA1* the most common genetic risk factor. Patients with DLB who carry *GBA1* mutations present with younger onset, more severe cognitive and motor dysfunction, higher rates of hallucinations and RBD and reduced survival [7–9]. In parallel, the *APOE ε4* allele, a significant risk factor for AD, is highly prevalent in DLB and is associated with more severe disease when in combination with *GBA1* mutations [9]. Together, *GBA1* and *APOE* represent key genetic modifiers that may shape biological disease expression in DLB.

[^18^F]FDG PET is an established supportive biomarker in DLB [10]. Conventional region-based or visual interpretation approaches incompletely capture the biological complexity arising from mixed pathology, motivating the use of quantitative and pattern-based analyses that better reflect network-level dysfunction arising from interacting pathologies. Classical [^18^F]FDG PET features include occipital hypometabolism and relative sparing of the posterior cingulate cortex, known as the cingulate island sign (CIS) [11]. Specificity can be vastly improved by advanced analyses such as the Scaled Subprofile Model/Principal Component Analysis (SSM/PCA), which allows identification of disease-related metabolic patterns across neurodegenerative diseases including Alzheimer’s disease (AD), PD and DLB [11–15].

Despite growing evidence that genetic factors influence clinical phenotype and prognosis in DLB, it remains unknown whether and how *GBA1* and *APOE* modulate [^18^F]FDG PET metabolic signatures. To address this, we investigated [^18^F]FDG PET metabolic features in a well-characterized cohort of AJ patients with DLB, stratified by *GBA1* mutation and *APOE ε4* status and their first-degree family members, who were included as a comparison cohort and to explore whether genetically at-risk individuals exhibited early metabolic alterations. We combined regional metabolic analysis, covariance-based disease pattern expression and multiclass machine learning modeling to test the hypothesis that genetic background shapes biologically meaningful [^18^F]FDG PET phenotypes in DLB.

## Methods

Study protocols were approved by the IRB Committees for Genetic Studies and institutional review board (IRB) of the TLVMC (numbers 0336-13-TLV and 0330-19-TLV), and conducted in accordance with the Declaration of Helsinki. The study adhered to international guidelines for clinical research, ensuring participant safety and data confidentiality. Written informed consent was obtained from all participants. In total 78 subjects were included between August 2019 until December 2023, of which 43 had DLB, 18 healthy controls (HC), and 17 relatives of the DLB patients.

Out of the included DLB patients, 14 (32.6%) were *GBA1* carriers (*GBA1(*+)). The most common variant was N370S [11], followed by 84GG [2] and E326K [1]. Two out of the 17 relatives were *GBA1* carriers (11.8%), one with N370S and one with the 84GG variant. Thirteen DLB subjects (30.2%) had at least one copy of the *APOE ε4* gene, of which two subjects were homozygotes. Three subjects were carriers of both *APOE ε4* and *GBA1*. A demographic overview of the included subjects can be seen in Table 1.

**Table 1:** Participant demographics (mean and standard deviation)

	**HC**	**REL**	**DLB**	
N	18	17	43	
Age (y)	73.9 (7.7)	47.4 (6.7)	71.2 (6.7)	
Sex (male)	44%	71%	88%	
Disease duration (y)	-	-	3.2 (2.3)	
MDS-UPDRS III	1.7 (2.6)	1 (1.1)	28.5 (12.0)	
MMSE	28.8 (1.0)	29.4 (0.7)	24.3 (4.7)	
MoCA	27.2 (2.3)	27.4 (2.8)	19.3 (5.6)	
GBA1(+), N	1 (6%)	2 (11.8%)	14 (32.6%)	
APOE ***ε4(+)***,*** N***	3 (17%)	-	13 (30.2%)	
GBA1(+) & APOE ***ε4(+)***,*** N***	0 (0%)	-	3 (7.0%)	
**Baseline Characteristics of DLB patients stratified by GBA1 and APOE ε4 Status**				
**Patients with DLB**	***GBA1 (*** **+** ***)***	***GBA1 (-)***	***APOE ε4 (+)***	***APOE ε4 (-)***
N	14	29	13	30
Age (y)	68.6 (7.8)	72.4 (5.8)	71.2 (6.4)	71.1 (6.9)
Sex (male)	93%	86%	85%	90%
Disease duration (y)	2.6 (1.6)	3.5 (2.6)	3.2 (2.3)	3.2 (2.4)
MDS-UPDRS III	25.4 (13.1)	30.0 (11.4)	25.4 (15.1)	29.8 (10.4)
MMSE	25.8 (3.7)	23.6 (5.0)	21.9 (6.1)	25.3 (3.6)
MoCA	21.8 (4.8)	17.9 (5.6)	18.0 (6.6)	19.8 (5.2)

Abbreviations: GBA1(+); GBA 1 carriers, GBA1(-); GBA 1 non carriers, APOE ε4(+); APOE ε4 carriers, APOE ε4 (-); APOE ε4 non carriers, REL; healthy relatives of DLB patients, MDS-UPDRS III; Movement Disorder Society-Unified Parkinson’s Disease Rating Scale Part III (Motor Examination), MMSE; Mini-Mental State Examination. MoCA; Montreal Cognitive Assessment

### Neurological evaluation

All participants underwent full neurological examination. An experienced neurologist obtained a detailed medical history, including symptoms and medications. Age at onset was defined as the onset of either cognitive decline or parkinsonism. All patients fulfilled the 2017 McKeith clinical criteria for possible or probable DLB [1], with criteria retrospectively applied to those recruited before 2017, and all had onset of motor symptoms less than one year prior to onset of cognitive decline. Motor symptoms were assessed using the Unified Parkinson’s Disease Rating Scale (UPDRS motor part III). Global cognitive function was assessed by the Mini-Mental State Examination (MMSE) [16] and by the Montreal Cognitive Assessment (MoCA) [17]. The presence of REM sleep behavior disorder (RBD) was assessed by the RBD Questionnaire (RBDQ) [18], and the existence of hallucinations, currently or in the past, was assessed by direct questions to the patients and the primary caregiver. Patients who were taking levodopa preparations remained on their usual dose. Patients receiving antipsychotic medications were excluded from this study.

Genotyping of founder GBA1 mutations was performed as previously described [19]. Briefly, patients were tested for p.Leu29AlafsTer18, c.115 + 1G > A, p.N409S, p.L483P, p.V433L, p.R535H, and 370Rec GBA1 variants, using the “Gaucher kit” (Catalog number 800672, Savyon Diagnostics, Israel, https://www.savyondiagnostics.com/product/nanochip-gaucher). The p.E365K and p.T408M GBA1 variants were genotyped using gene-specific TaqMan assay, followed by Sanger sequencing of PCR products, to confirm carriers. GBA1 pathogenic variants were divided into three: 1. Non-Gaucher disease (GD) causing (p.E365K and p.T408M), 2. Variants associated with mild Gaucher disease (p.N409S and p.R535H) 3. Variants associated with severe Gaucher disease (p.Leu29AlafsTer18, c.115 + 1G > A, and p.V433L). Existence of the LRRK2 G2019S mutation was also examined. All samples were genotyped for rs429358 and rs7412 using TaqMan assays (C___3084793_20 and C____904973_10, respectively; Applied Biosystems) to establish APOE haplotypes. APOE ε2- and ε4-positive carriers were subsequently confirmed by PCR amplification of a specific fragment using the following primers: forward 5′-GGCACGGCTGTCCAAGGAGCT-3′ and reverse 5′-GCCCCGGCCTGGTACACTGC-3′, followed by Sanger sequencing.

### FDG PET acquisition, preprocessing and analyses

Seventy-four subjects were scanned on a GE Discovery MI camera, and four on a GE Discovery 690, and the images were reconstructed with the VPFXS/VPFX algorithm. Two subjects (one DLB patient and one relative) that were scanned on the Biograph Vision were excluded due to technical reasons.

All [^18^F]FDG PET scans were spatially normalized to an [^18^F]FDG PET template in MNI152 space using SPM12, followed by 8 mm Gaussian smoothing [20].

#### Regional analysis

For the regional analysis, the [^18^F]FDG PET scans were intensity normalized with global mean normalization. A gray matter mask consisting of regions of interest (ROI) included in the AAL2 atlas as implemented by the WFU Pickatlas tool was used to compute the overall uptake within the brain [21–23]. The mask/atlas is visualized in Fig. 6 in Appendix A. Thereafter, the mean uptake in regions of the AAL2 atlas were considered and differences in ROI uptake were compared on a group level, i.e., considering GBA1 carriers vs. non-carriers and *APOE ε4* carriers vs. non-carriers, respectively. Additionally, the cingulate island sign (CIS) was computed in the same manner as in Gjerum et al. [24]. There, the CIS was computed separately for each hemisphere by considering the mean uptake in the posterior cingulate cortex over a combined region of the precuneus and cuneus. The overall CIS value was then considered as the average of the left and right CIS. A higher CIS indicates a relatively more preserved Posterior cingulate cortex (PCC), a DLB-specific signature. For interpretability, all regional values including the CIS were transformed into z-scores with respect to the HC group.

#### Metabolic disease patterns

We considered pattern expressions in our data with respect to the Alzheimer’s disease related pattern (ADRP), Parkinson’s disease (PDRP) and Dementia with Lewy Bodies related patterns (DLBRP). These metabolic patterns were determined in independent data and published previously [12, 25, 26]. For this analysis, the [^18^F]FDG PET scans were intensity normalized with the Scaled Subprofile Model (SSM) method, where the mask and grand mean profile was given as a byproduct of the pattern extraction. Thereafter, the raw pattern scores were extracted as the dot product between a scan and the disease pattern, and finally the scores were converted into z-scores based on the mean and standard deviation of the HC group. In-house written script was used for this method (for a full methodological description, see Meles et al. [27]).

#### Machine learning analysis

We trained a Generalized Matrix Learning Vector Quantization (GMLVQ) using an external cohort of patients imaged at the University Medical Center Groningen, as previously proposed in van Veen et al. [28]. The cohort included 56 HC, 23 DLB, 38 PD and 53 AD patients, with imaging protocols described previously [14, 27, 29, 30]. We considered mean uptake in regions of the AAL2 atlas, merging all regions of the Cerebellum into one feature, and considering the average of corresponding pairs of left and right regions. This resulted in mean uptake values from 48 regions in total. The uptake values were z-scored to the mean and SD of the healthy controls of the UMCG, and site effects between the two centers were harmonized by applying Iterated Relevance Matrix Analysis on the corresponding HC cohorts [31]. Here, the ten oldest healthy relatives from Tel Aviv were added to the HC/harmonization cohort. Thereafter, we trained a center-harmonized GMLVQ model to classify HC-PD-DLB-AD, where the training set was randomly up-sampled to an equal number of samples per class. We used the cohort acquired in Tel Aviv as a test set, and evaluated the position in the model space with respect to genetic status. Full details of the training procedure are provided in Appendix A. In particular, we evaluated whether *GBA1* non carriers tend more toward AD compared to *GBA1* carriers, and whether *GBA1* carriers tend more toward idiopathic PD.

#### Statistical methods

We computed group-level differences in the ROIs, disease patterns and machine learning model, considering all *GBA1* carriers vs. *GBA1* non carriers and *APOE ε4* carriers vs. *APOE ε4* non carriers. Differences in clinical scores between the groups were evaluated with ANCOVA with age and disease duration as covariates. Age and disease duration were compared between groups without covariate adjustment. Due to the small number of subjects, differences between specific GBA1 variants were not examined. Dual carriers were included in the primary analyses. A separate sensitivity analysis excluding dual carriers was performed and did not significantly alter the results, therefore dual carriers were retained in the main analyses. For the AAL2-ROI analysis, we applied ANCOVA with age and disease duration as covariates, and Benjamini-Hochberg correction for multiple comparisons. We tested the cingulate island sign, disease pattern scores, and outcome from the machine learning model in a hypothesis-driven manner, with a Kolmogorov-Smirnov test with uncorrected p-values. This choice was made since we preferred not to make parametric assumptions about the distributions, and in addition to detecting differences in central tendencies, the K-S test is sensitive to any type of difference in distribution, e.g. multi-modality or variance. Clinical correlations were evaluated with Spearman’s rank coefficient. We used the Python sklvq package [32] for implementation of the GMLVQ model, and Scipy v1.16.0 [33] and Pingouin v0.5.5 [34] for realization of the statistical tests.

## Results

### Participants

#### Clinical characteristics

There were no significant differences in age or disease duration between *GBA1* carriers and non carriers (*p* = 0.09 and *p* = 0.24, respectively), or *APOE ε4* carriers and non carriers (*p* > 0.9 for both). The *APOE ε4* carrier group had lower MMSE scores than *APOE ε4* non carrier group (*p* = 0.03), while there was no significant difference in MoCA and MDS-UPDRSIII, or for any of the cognitive or motor scores comparing the *GBA1* groups (*p* > 0.1).

### Regional analysis

#### AAL2 regions

Multiple brain regions showed differences between the groups, however, none remained significant after correction for multiple comparisons.

#### Cingulate island sign

**Fig. 1:**
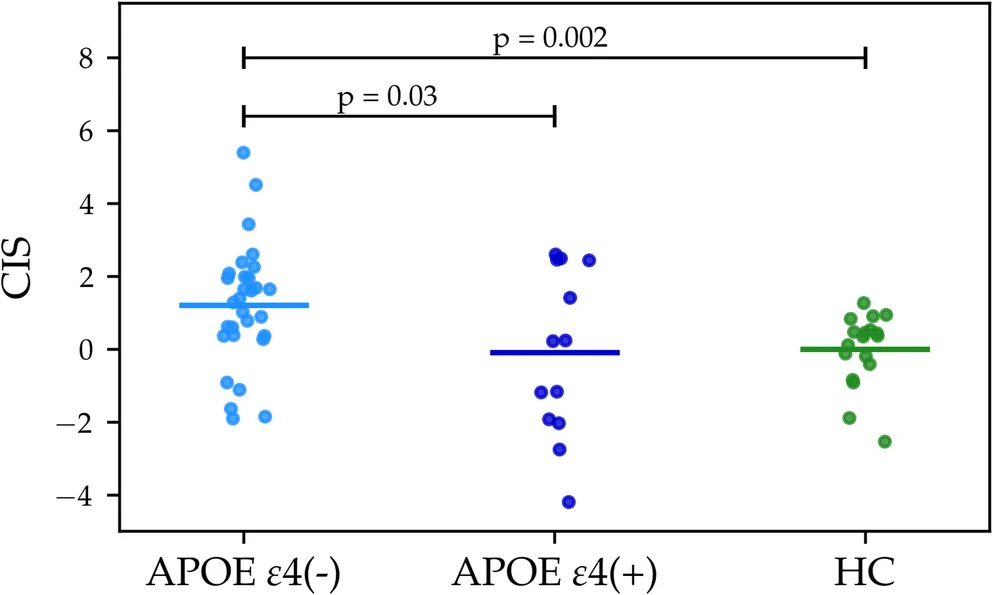
Cingulate island sign z-scores for *APOE ε4(*-) vs. *APOE ε4(+)*, together with HC as a reference

The *APOE ε4 *non carrier group displayed a significantly **higher** cingulate island sign than the *APOE ε4 *carrier group (median 1.35 vs. 0.23, *p* = 0.03, see Fig. 1). The CIS z-scores for HC are also included here as a reference. There was no significant difference in CIS between *GBA1 *carriers and *GBA1 *non carriers. The difference in CIS between *APOE* groups remained significant also when correcting for age and disease duration (*p* = 0.04 with ANCOVA).

### Pattern scores

**Fig. 2:**
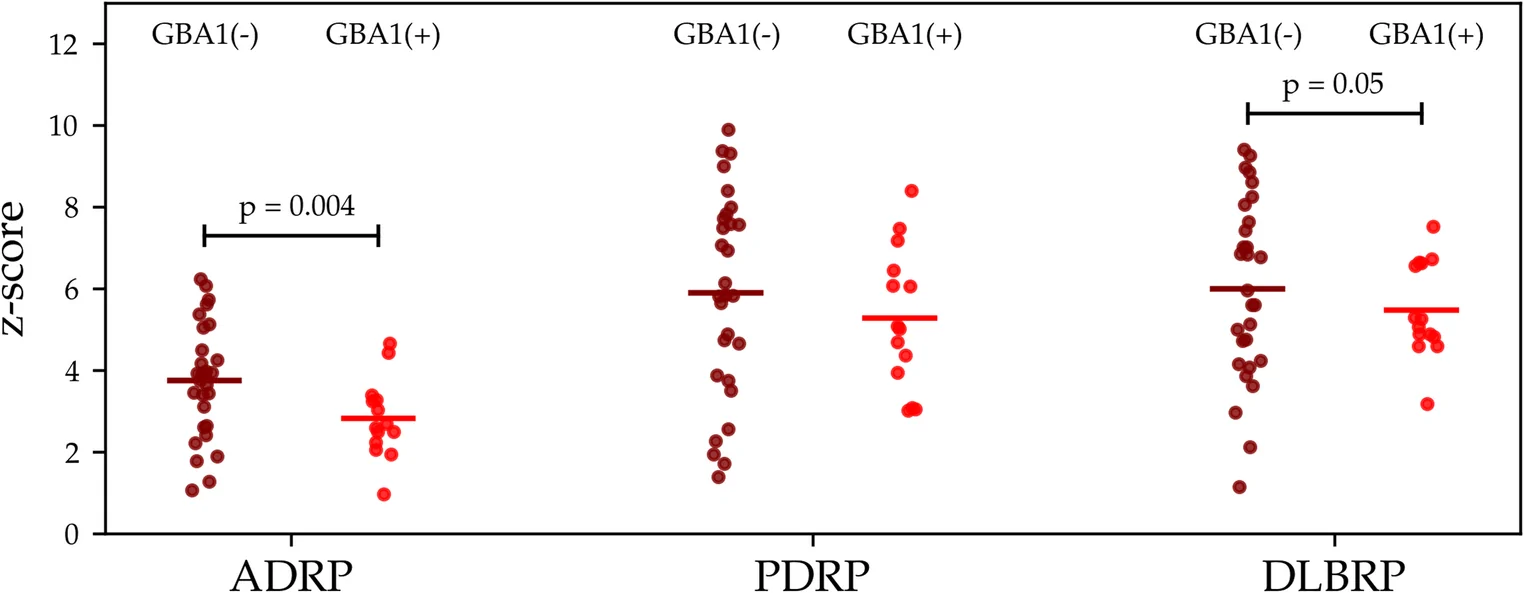
Disease pattern scores for *GBA1 (-)*; (GBA non carrier) and *GBA1 (+);* (GBA carrier) DLB patients

**Fig. 3:**
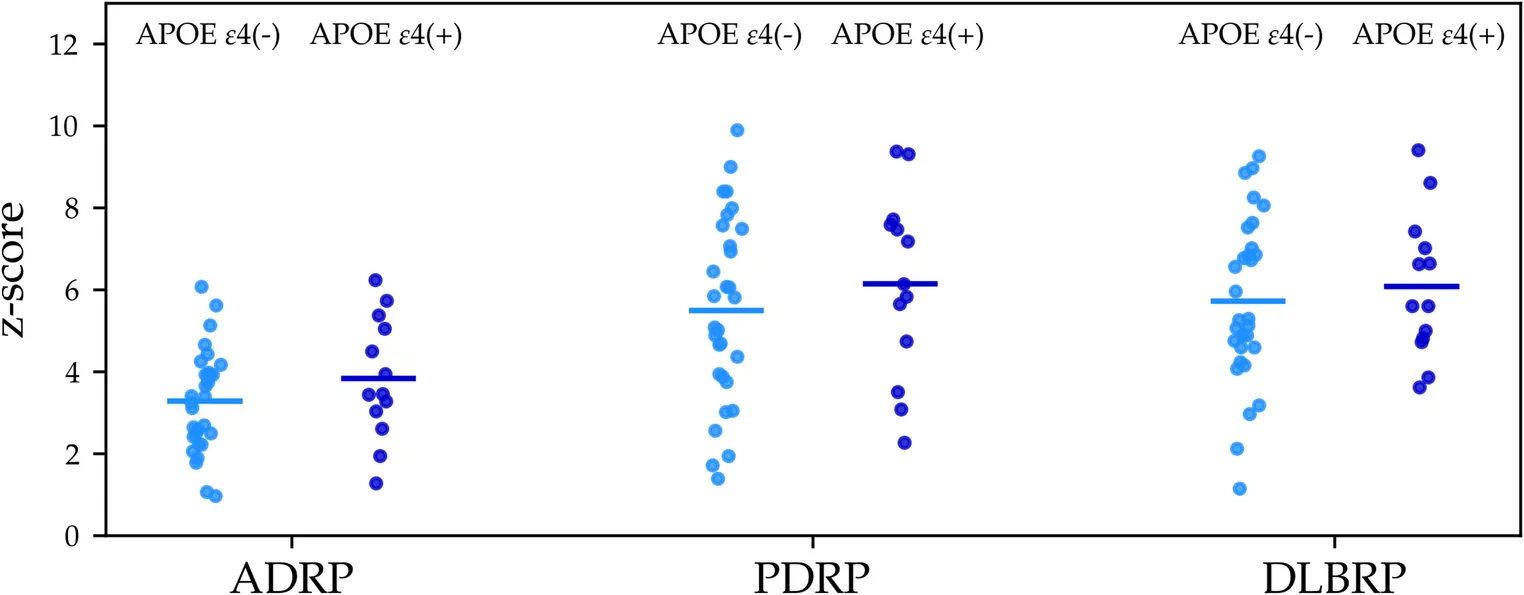
Disease pattern scores for *APOE ε4 (+);(APOE ε4* carrier) vs. *APOE ε4 (-);* (APOE ε4 non carrier) DLB patients

The DLB patients displayed a significantly higher DLBRP z-score than HC (5.8 ± 1.9 vs. 0.0 ± 1.0, *p* < 0.0001). The *GBA1* non-carrier DLB patients displayed a higher expression and higher variance in the ADRP than the *GBA1* carrier group (median and IQR 3.9 [2.7, 4.5] vs. 2.7 [2.3, 3.3], *p* = 0.004). The same trend was seen for the PDRP and DLBRP patterns, where only the difference in DLBRP expression was significant (median and IQR 6.0 [4.2, 7.6] vs. 5.1 [4.8, 6.6], *p* = 0.05). For PDRP scores the corresponding medians were 5.1 [4.1, 6.4] and 5.9 [3.9, 7.7] (*p* = 0.25). Notably, both 84GG carriers had among the highest DLBRP z-scores in the *GBA1 *carrier group, with z-scores of 6.6 and 6.7, respectively. The distributions in pattern z-scores for the *GBA1* groups can be seen in Fig. 2. There were no differences in the pattern scores between the *APOE ε4* groups (see Fig. 3). Notably, the three subjects with the lowest DLBRP scores were all non-carriers for both *GBA1* and *APOE ε4*.

### GMLVQ analysis

**Fig. 4:**
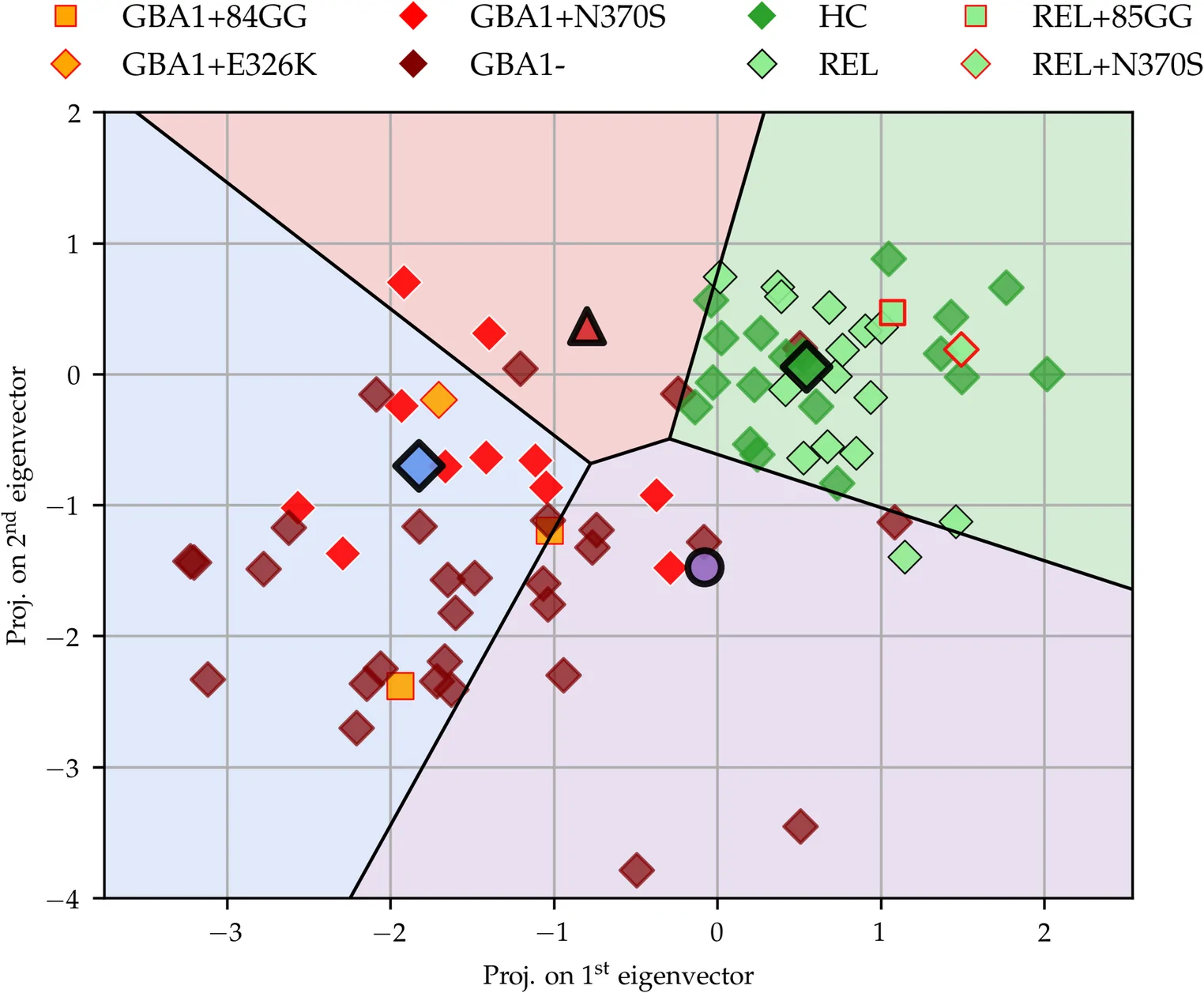
Trained GMLVQ model space, with the Tel Aviv cohort projected in as a test set. The HC decision space corresponds to the green area (right), AD purple (bottom), PD red (middle/upper), and the DLB decision space is blue to the left of the map. Corresponding prototypes (typical examples of each class) are shown as larger markers with a thick black border. Each axis in the plot can be interpreted as a linear combination of weighted ROIs. Each corresponding, orthogonal vector contributes to separating the four classes in the internal model space

The trained GMLVQ model space, with the HC, relatives and DLB patients from Tel Aviv projected in as a test set is shown in Fig. 4. The model converged into a three-dimensional internal representation after training, where the most important directions of the decision space can be visualized in 2D. While the *GBA1 *non carrier subjects had a higher misclassification rate for AD (*N* = 9, 31% for *GBA1 (-) *vs. *N* = 3, 21% for *GBA1 (+)*, and *GBA1* carrier with PD (*N* = 2, 14% for *GBA1 (*+) vs. *N* = 2, 7% for *GBA1 (-)*, the difference was not significant (*p* = 0.71). Overall, 60% of the DLB patients were correctly classified, with AD being the most common class a sample was confused with (*N* = 12, 28%), followed by PD (*N* = 4, 9%) and HC (*N* = 1, 2%). 94% of HC or healthy relatives were correctly classified as such, while two samples (6%) fell into the beginning regions of the AD decision space. The two relatives who were *GBA1* carriers were well within the healthy space and therefore, neither had an indication of an elevated risk of developing DLB. The ROC-AUC for HC vs. DLB was 0.98. Since it is nontrivial to measure ROC-AUC in a multi-class problem where the test distribution only contains some of the classes, it was measured by the predicted probabilities for the HC class vs. all other classes. Similarly, the sensitivity/specificity for the DLB class was 0.60/1.00, and for the HC class 0.94/0.97.

Considering the distance to each of the prototypes in the model, *GBA1 *carrier DLB patients tended more towards PD patients than those who were *GBA1 *non carrier (*p* = 0.01). *GBA1 *non carrier patients overall fell further away from the HC prototype (*p* = 0.03), which in the model can be seen both as outliers in AD space, but also the group of patients placed very far into the DLB space (leftmost area of the model space, representing a severe metabolic expression). *GBA1 *carrier patients also had a tighter distribution around the DLB prototype compared to *GBA1 *non carrier (*p* = 0.05), and the *GBA1* non carrier group were overall placed lower along the second eigenvector of the model (*p* = 0.002), representing a higher similarity to AD compared to PD. The *GBA1* carrier group was also more clustered than *GBA1* non carriers considering the pairwise distances within the groups (*p* = 0.004).

*APOE*-status did not have a significant influence on the position of the DLB patients in model space (see Fig. 7 in Appendix A). Similarly, age had no significant impact on the model space (see Fig. 8 in Appendix A), both measured as the distance between the DLB patients and each of the prototypes (*p* > 0.20).

The CIS correlated with distance to the DLB prototype in the GMLVQ model (*r* = −0.37, *p* = 0.01), but not with distance to AD prototype (*r* = 0.26, *p* = 0.09).

### Clinical correlations

**Fig. 5:**
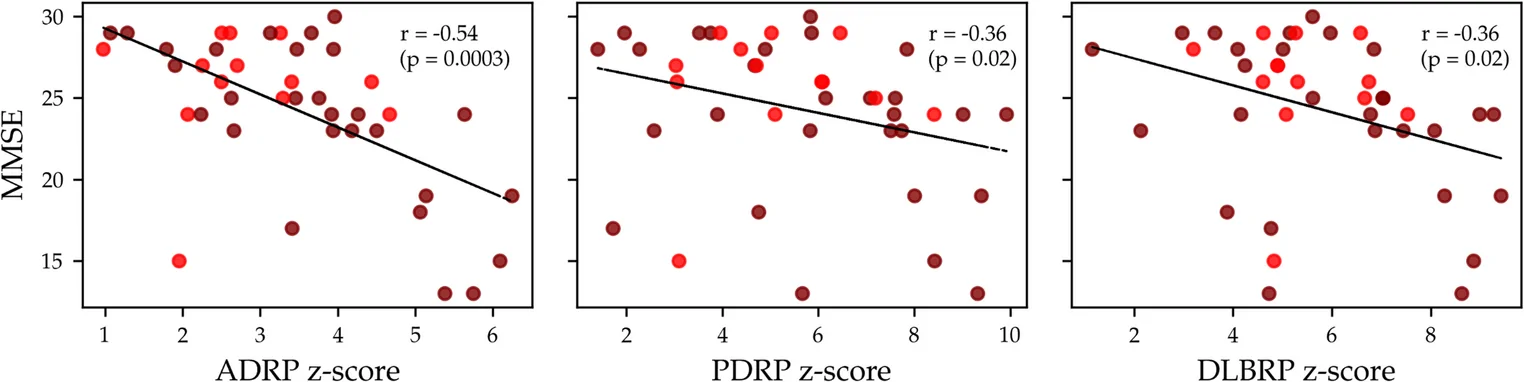
MMSE correlated negatively with the disease pattern scores. *GBA1* (+); (*GBA1* carrier) subjects are plotted in red, *GBA1* (-); (*GBA1* non carrier) in maroon

**Fig. 6:**
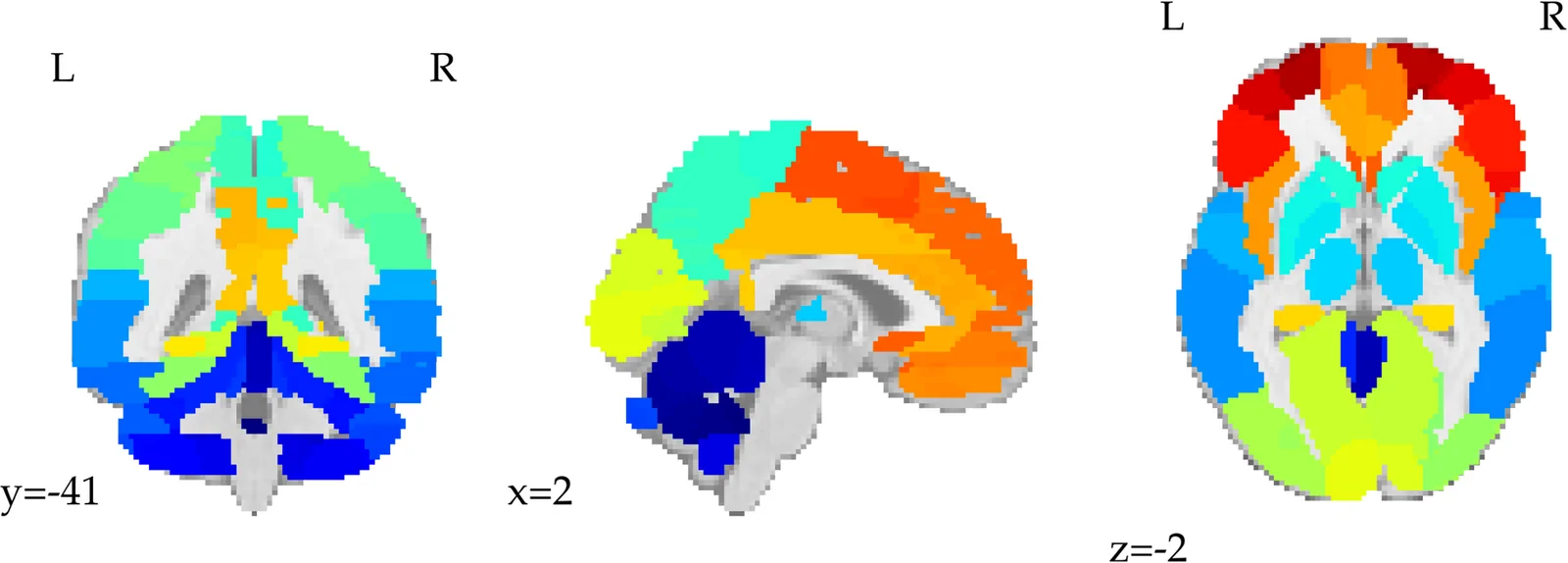
Regions of the AAL2 atlas used as a mask and for the ROI-based analysis

**Fig. 7:**
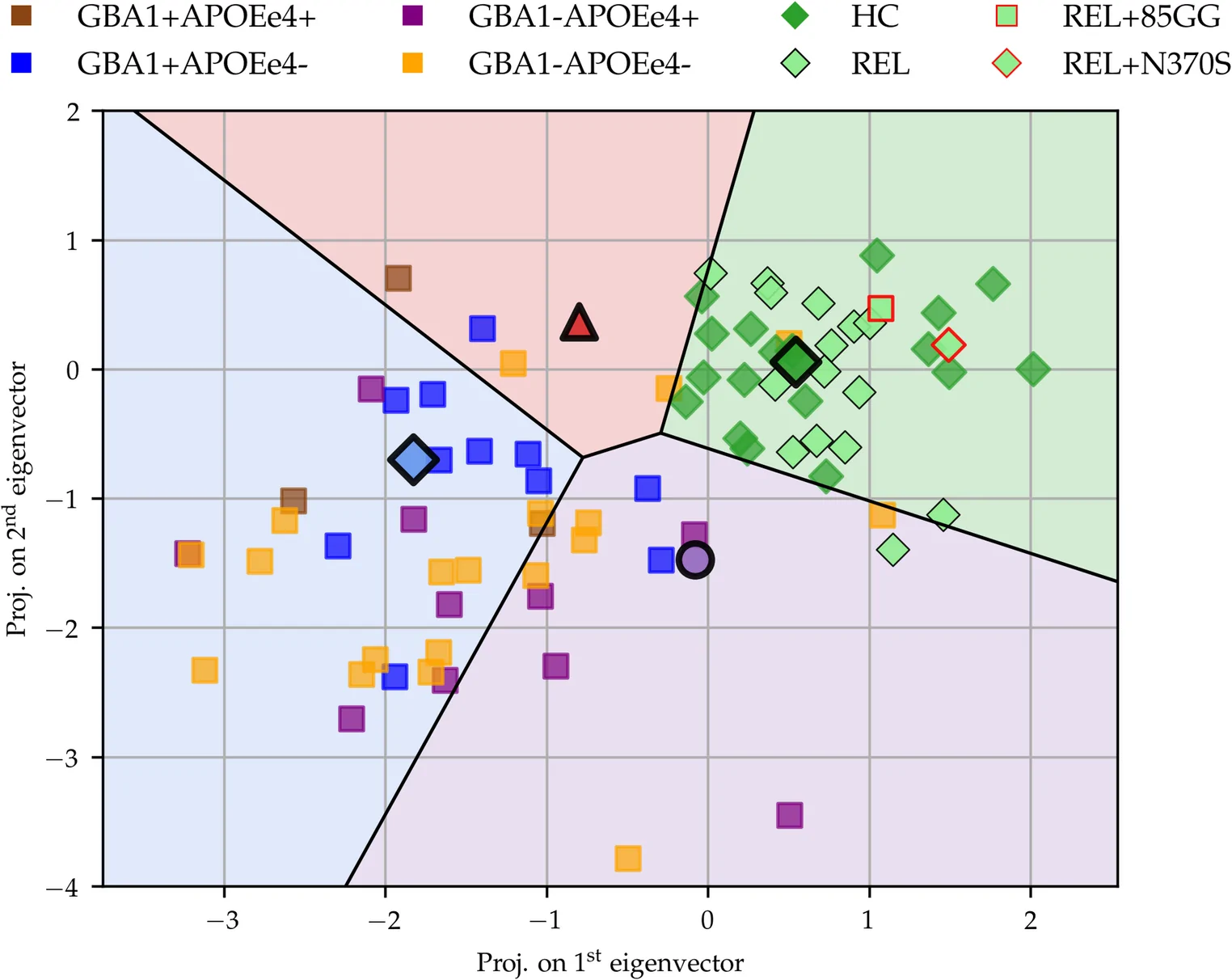
Tel Aviv DLB patient cohort projected into the GMLVQ model space, color coded by *GBA1* and APOE status. While the *GBA1(*+) patients displayed a more homogeneous pattern compared to *GBA1(*-), APOE status did not have a significant effect

**Fig. 8:**
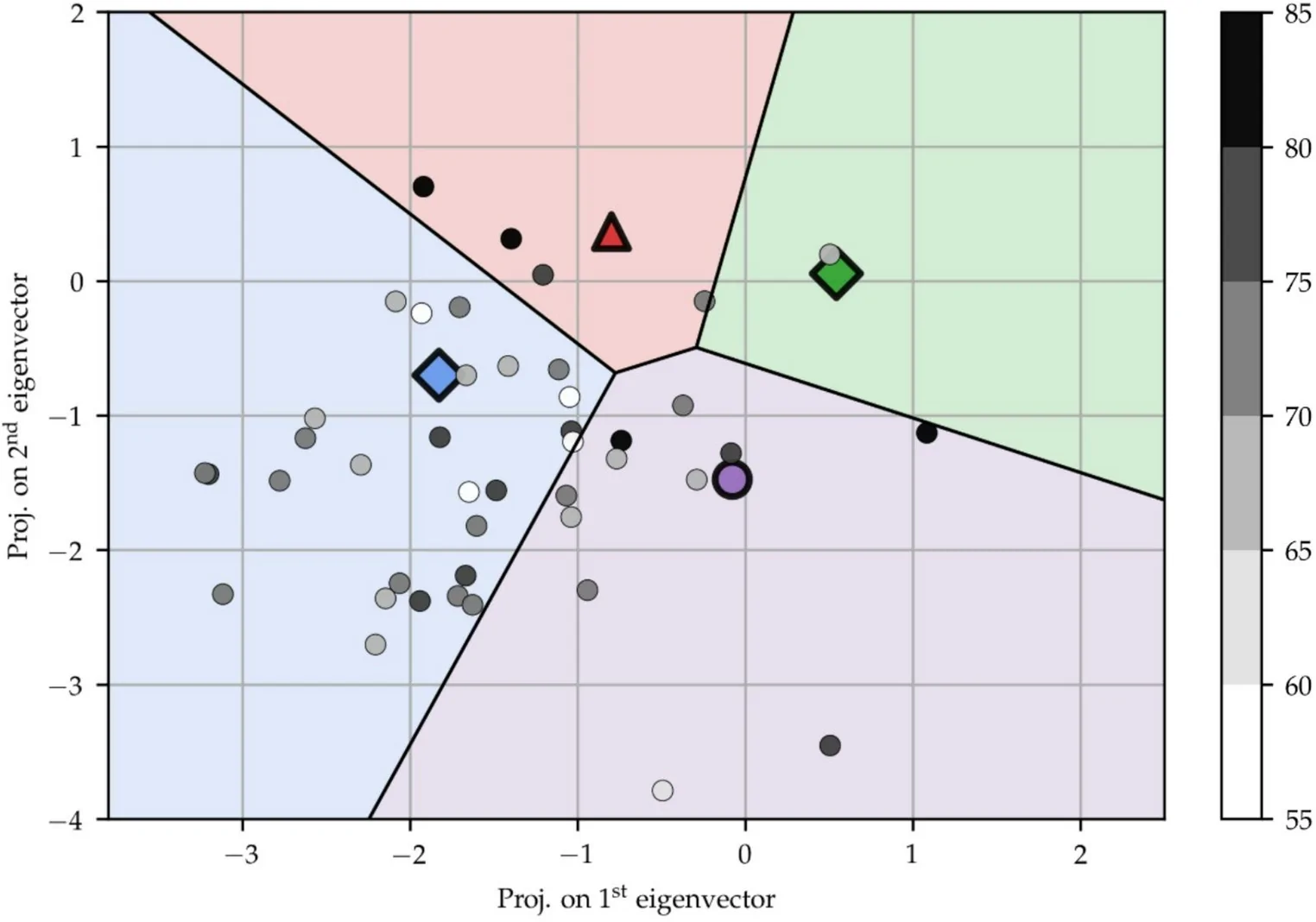
Tel Aviv DLB patients projected into the GMLVQ model space, color coded by age. There was no significant correlation between age and distance to any of the prototypes, or placement on the x- or y-axis

MMSE score correlated negatively with all disease pattern scores (*r* = −0.54, −0.36, −0.36, *p* < 0.001, *p* = 0.02, *p* = 0.02) for the ADRP, PDRP and DLBRP z-scores, respectively. MoCA scores also negatively correlated with disease pattern z-scores (*r* = −0.47, −0.34, −0.33, *p* = 0.002, 0.03, 0.04). Higher cingulate island sign correlated with higher DLBRP z-scores (*r* = 0.33, *p* = 0.03), correlated weakly with PDRP z-scores (*r* = 0.28, *p* = 0.07), but not with ADRP z-scores (*r* = −0.04, *p* = 0.82) (Fig. 5).

For the *APOE ε4* non carrier group the cingulate island sign correlated negatively with years of education (*r* = −0.49, *p* = 0.006), but was not related to MMSE or MoCA scores (*p* = 0.44 and 0.38, respectively). For the *APOE ε4* carrier group, the correlation between cingulate island sign and MMSE was significant (*r* = 0.61, *p* = 0.04). Neither group had a significant correlation between disease duration and cingulate island sign (*p* = 0.24 and *p* = 0.76, respectively).

UPDRS-III scores did not correlate with any of the three disease pattern scores (*p* ≥ 0.1), but did correlate with disease duration (*r* = 0.41, *p* = 0.007). There was no significant correlation between disease duration and DLBRP score (*p* = 0.13).

## Discussion

In this study, we show that common genetic modifiers in DLB are associated with biologically meaningful differences in [^18^F]FDG PET metabolic phenotypes across regional, network-based, and machine-learning analyses. *APOE ε4* primarily influenced posterior cingulate vulnerability reflected in attenuation of the cingulate island sign, whereas *GBA1* mutation status was associated with distinct global metabolic profiles, with *GBA1* carriers exhibiting a more homogeneous alpha-synuclein-associated pattern and *GBA1* non-carriers demonstrating greater heterogeneity and increased Alzheimer-like metabolic drift.

### Cingulate island sign

In this cohort of patients with DLB, *APOE ε4* carriers exhibited a significantly lower cingulate island sign (CIS) compared to non-carriers, indicating reduced preservation of posterior cingulate metabolism relative to the precuneus and cuneus. The CIS has been consistently linked to a “pure DLB” metabolic phenotype, with relative sparing of posterior cingulate cortex in the context of marked occipital hypometabolism [11]. A diminished CIS in *APOE ε4* carriers therefore suggests a weaker DLB-specific metabolic signature.

This interpretation aligns with the established role of *APOE ε4* in promoting amyloid-β and downstream tau aggregation [35], both of which are common co-pathologies in DLB and vary widely between individuals [36, 37]. Recent multimodal PET evidence shows that tau accumulation in the PCC directly drives CIS decline and correlates with cognitive impairment in Lewy body disease [38]. As tau burden and associated PCC hypometabolism increases, [^18^F]FDG PET patterns shift toward those typical of AD — weakening the metabolic contrast that traditionally supports accurate differentiation.

These findings have important diagnostic implications. [^18^F]FDG PET interpretation in DLB relies on metabolic dissociation between the PCC and occipital cortex. *APOE ε4* carriers, who have been reported to show higher rates of AD co-pathology [39], may thus be more vulnerable to misclassification as AD when CIS is reduced. This challenge is amplified in earlier disease stages when occipital hypometabolism remains subtle and the diagnostic value of the CIS is greatest [40]. Furthermore, *APOE ε4* has been associated with a more aggressive cognitive trajectory and shorter survival in DLB [8, 35]. Our imaging findings support the hypothesis that *APOE ε4* carriers express an AD-shifted metabolic phenotype, consistent with their poorer prognosis and clinical heterogeneity in cognitive profiles.

### Pattern scores

DLB patients in this cohort demonstrated the expected elevation in the DLB-related metabolic pattern (DLBRP), consistent with the characteristic occipital and parietotemporal hypometabolism described in prior covariance-based analyses of synucleinopathies [11, 13, 41]. Importantly, however, stratification by *GBA1* genotype revealed meaningful metabolic differences: *GBA1* non-carriers expressed significantly higher ADRP scores and greater variability in ADRP, PDRP and DLBRP, whereas *GBA1* carriers expressed a more uniform metabolic phenotype implying a more consistent pathological process. While there is partial topographic overlap between the ADRP and PDRP, particularly in parietal hypometabolism and relative sparing or hypermetabolism of subcortical and cerebellar regions, these patterns are defined by distinct multiregional covariance structures and have been consistently shown to reflect disease-specific network dysfunction across neurodegenerative syndromes [11, 13, 42].

These findings are biologically plausible. *GBA1* mutations are associated with increased alpha-synuclein pathology, which is often more prominent and less confounded by concurrent AD pathology [43, 44]. This model aligns with clinical observations in AJ-DLB, where *GBA1* carriers show younger onset, more pronounced visual hallucinations, greater motor burden and increased REM sleep behavior disorder [7, 9, 45], symptoms often associated with alpha-synuclein pathology.

In contrast, *GBA1* non-carriers who often carry no identifiable synucleinopathy-risk variant—may exhibit greater metabolic heterogeneity, potentially reflecting a broader range of underlying pathologies, including AD co-pathology. This could contribute to greater ADRP expression, consistent with our findings and supported by neuropathological series showing increased AD co-pathology in *GBA1*-negative DLB [2, 43].

The absence of significant differences in pattern scores between *APOE ε4* carriers and non-carriers is also informative. Although *APOE ε4* modulated CIS expression, its influence on global pattern expression appears limited, suggesting that the CIS may be more sensitive to metabolic changes associated with AD co-pathology than ADRP scores themselves. This interpretation is supported by Nakamura et al. [38], who demonstrated that tau-driven PCC hypometabolism reflects AD burden more directly than regional covariance patterns.

### GMLVQ Machine learning analysis

The machine-learning analysis provides convergent evidence that genetic background shapes metabolic phenotype in DLB. Using a center-harmonized GMLVQ classifier trained on an external cohort [28, 31], we projected our cohort into the trained classification space and observed clear genotype-related trends.

Specifically, *GBA1* non-carriers scattered farther from both the DLB and healthy-control prototype and showed greater displacement toward lower regions of the classification map, consistent with their higher ADRP scores and broader metabolic heterogeneity. Conversely, *GBA1* carriers clustered more tightly around the DLB prototype and displayed a modest drift toward the PD prototype, in line with an alpha-synuclein-dominant metabolic profile and prior clinical evidence linking *GBA1* mutations to a PD-like phenotype. These findings parallel covariance-based pattern analyses in synucleinopathies [13, 42] and demonstrate the added value of multiclass classification approaches over single-pattern scores.

The finding that *APOE ε4* status did not significantly influence placement in the GMLVQ model underscores the idea that APOE’s metabolic impact in DLB is regionally specific (i.e., PCC vulnerability reflected in CIS) rather than globally pattern-shifting.

Machine learning approaches such as GMLVQ are particularly well suited for detecting subtle metabolic signatures across disease spectra. By incorporating multiple classes simultaneously, the model captures relative disease similarity and shifts reflecting mixed pathology and network-level vulnerability. Our model, trained on external data, had a 60% sensitivity for the Tel Aviv DLB patients. This is in line with previous research highlighting the metabolic heterogeneity in DLB and that misclassifications for DLB are frequent in multi-class models. For example, van Veen et al. [28] reported a cross-validation sensitivity of 58% for DLB when considering the same four classes as in our current work. Our findings position GMLVQ as a promising adjunct to traditional [^18^F]FDG PET analysis for biologically informed patient stratification in DLB. The map of the classification space also enables appreciation of the significant heterogeneity of the DLB spectrum.

Taken together, the three analytical approaches highlight different aspects of the metabolic phenotype in DLB. The regional analysis showed that APOE ε4 primarily affected the cingulate island sign, suggesting greater vulnerability of the posterior cingulate cortex and supporting its association with AD-like metabolic changes. In contrast, both the covariance-based disease pattern analysis and the machine learning model pointed to an effect of *GBA1* on the overall metabolic phenotype. Across both analyses, *GBA1* carriers showed a more homogeneous DLB/PD-like metabolic profile, whereas *GBA1* non-carriers were more heterogeneous and showed greater expression of AD-like metabolic patterns. The lack of a clear APOE ε4 effect in the global analyses suggests that APOE ε4 may have a more localized effect on posterior cingulate metabolism, whereas *GBA1* is associated with broader differences in whole-brain metabolic organization. Together, these findings suggest that APOE ε4 and GBA1 influence different aspects of the metabolic phenotype in DLB.

### Clinical correlations

Clinical correlations in this cohort reinforce the biological validity of the imaging findings. Cognitive scores (MMSE, MoCA) correlated negatively with ADRP, PDRP and DLBRP expression scores, consistent with prior evidence that greater expression of disease-related metabolic networks is associated with increased cognitive impairment and disease burden, rather than representing a simple linear measure of disease stage [15, 46, 47].

Motor severity (UPDRS III) correlated with disease duration but not with metabolic patterns, possibly due to the known dissociation between motor progression and cortical metabolic involvement in DLB [45, 46, 48, 49]. *GBA1* or *APOE ε4* status did not significantly influence clinical correlations. Perovnik et al. [15] reported a similar correlation between DLBRP expression and motor severity to that observed in our cohort (*r* = 0.26 vs. *r* = 0.25, respectively). The statistical significance observed in their study is likely explained by their larger sample size, which provided greater power to detect this relatively modest association.

Our study has several limitations. First, our sample size is small, in particular when splitting up the patient sample into subgroups based on genetic status. For example, we were not able to consider specific variants of the mutations in our analysis. Furthermore, our data did not allow for a mechanistic study due to the lack of additional metabolic biomarkers. While the groups did not differ in age or disease duration, the *APOE ε4* carrier group had a significantly lower MMSE than the *APOE ε4* non carriers (*p* = 0.03). Therefore, we cannot exclude the possibility that the *APOE* carriers had a more severe disease, which may have contributed to the observed metabolic differences. Given the close relationship between cognitive impairment and cerebral metabolism, it is not possible to disentangle these effects unambiguously. Nevertheless, the difference in CIS between *APOE* groups remained significant when applying an alternative statistical test (ANCOVA) correcting for age and disease duration (*p* = 0.04). Finally, each investigated group showed significant variance in the investigated metabolic quantities, and no metabolic signatures that would accurately determine genetic status on an individual level were identified.

In conclusion, in this study we examined how common genetic factors influence [^18^F]FDG PET metabolic patterns in dementia with Lewy bodies. We found that *APOE ε4* carriers showed attenuation of the cingulate island sign, suggesting increased vulnerability of the posterior cingulate cortex to AD like metabolic change and reduced preservation of a characteristic DLB-specific metabolic feature. In contrast, *GBA1* mutation carriers demonstrated a more homogeneous metabolic phenotype, consistent with a more alpha-synuclein-predominant metabolic phenotype, while *GBA1* non-carriers showed greater metabolic heterogeneity and increased expression of Alzheimer-like metabolic patterns.

Our findings demonstrate that regional metabolic markers, covariance-based pattern analysis, and multiclass machine-learning approaches provide complementary information. In particular, the cingulate island sign captured *APOE*-related effects that were not reflected in global pattern expression, whereas multiclass modeling highlighted relative disease similarity and phenotypic heterogeneity that were not apparent from single-pattern metrics alone.

Understanding how genetic background shapes metabolic phenotypes in DLB is important for improving diagnostic specificity and biological stratification. Incorporating genetic information with advanced [^18^F]FDG PET analyses may help identify clinically meaningful subgroups and inform patient selection for clinical trials, particularly those targeting Alzheimer co-pathology.

## Data Availability

The datasets generated during and/or analysed during the current study are available from the corresponding author on reasonable request.

## Additional details of machine learning analysis

This work used the same set of patients as in van Veen et al. (2022). Since this work used regional uptake values instead of PCA-based features, no patients had to be held out for the PCA application. Since the UMCG data set contained a few patients with young onset neurodegeneration, we excluded patients from the disease groups who were younger than 50 in the training set. Healthy controls younger than 54 were also excluded, to create a HC group that was roughly age-matched with the Tel Aviv cohort. For harmonization of center effects, we used a merged group of healthy controls and relatives from the Tel Aviv cohort, excluding relatives younger than 45 (resulting in a merged group of *N* = 28, age 65.8 ± 13.1). The IRMA method required four iterations (corresponding to a four-dimensional subspace) to remove notable center effects in the harmonization cohorts, with an initial balanced cross-validation accuracy of BAC = 0.93 when determining the center of origin in the harmonization cohort. The trained model had two dominant eigenvectors, and a third non-zero eigenvector contributing mildly to the model space (eigenvalue 0.13). While we display the model space in a two-dimensional plot, the distances when comparing groups were computed using all contributing directions. The center harmonization overall improved test performance vs. an uncorrected model (sensitivity 0.51 for DLB, 0.91 for HC/REL and ROC-AUC 0.97 for HC vs. DLB in the uncorrected model, with the same numbers being 0.60, 0.94 and 0.98 for the center-corrected model). Seven Tel Aviv relatives were not included in the harmonization cohort due to their young age, and all of these (100%) were correctly classified as healthy in the trained model. Since the Tel Aviv HC and some of the relatives were used to harmonize the data space, only the DLB patients and seven held out REL subjects can be considered a strict test set, since the formerly mentioned group was implicitly included in the model training via the harmonization step. Because the aim of this analysis was not diagnostic validation but characterization of the metabolic phenotype of the Tel Aviv DLB cohort, we believe this limited overlap in the harmonization step is unlikely to materially influence the conclusions. The REL+N370S subject was not included in the harmonization cohort while the REL+84GG subject was (Table 2).

**Table 2:** Overview of GMLVQ training data (UMCG cohort)

Group	*N*	Age (y) (S.D)	Sex (M)	Disease duration (S.D)
HC	56	64.5 (5.8)	61%	-
PD	38	67.1 (7.7)	71%	5.9 (5.5)
AD	53	67.0 (7.1)	60%	3.0 (3.1)
DLB	23	72.0 (7.5)	78%	2.3 (1.1)
